# Light-Driven Energy and Charge Transfer Processes between Additives within Electrospun Nanofibres

**DOI:** 10.3390/molecules28124857

**Published:** 2023-06-19

**Authors:** Reeda Mahmood, Tristan Mananquil, Rebecca Scenna, Emma S. Dennis, Judith Castillo-Rodriguez, Bryan D. Koivisto

**Affiliations:** Department of Chemistry and Biology, Toronto Metropolitan University, 350 Victoria St., Toronto, ON M5B 2K3, Canada

**Keywords:** electrospinning, FRET, energy transfer, organic dyes, nanoparticles, monoaxial and coaxial nanofibres, polymers

## Abstract

Electrospinning is a cost-effective and efficient method of producing polymeric nanofibre films. The resulting nanofibres can be produced in a variety of structures, including monoaxial, coaxial (core@shell), and Janus (side-by-side). The resulting fibres can also act as a matrix for various light-harvesting components such as dye molecules, nanoparticles, and quantum dots. The addition of these light-harvesting materials allows for various photo-driven processes to occur within the films. This review discusses the process of electrospinning as well as the effect of spinning parameters on resulting fibres. Building on this, we discuss energy transfer processes that have been explored in nanofibre films, such as Förster resonance energy transfer (FRET), metal-enhanced fluorescence (MEF), and upconversion. A charge transfer process, photoinduced electron transfer (PET), is also discussed. This review highlights various candidate molecules that have been used for photo-responsive processes in electrospun films.

## 1. Introduction

Polymeric materials doped with photoactive materials have widespread applications as sensing materials [1], photovoltaic devices [2], drug delivery vehicles [3], coatings, and films [4,5] and can also be used in biomimicry [6,7]. In addition to the variability imparted by the polymer composition, the function can be extended by incorporating additives such as dye molecules, metal nanoparticles, and quantum dots because they have the additional advantage of promoting energy transfer processes [8,9]. Yet, another advantage of polymeric materials is seen in the variety of processing methods, including spin coating, thermal spraying, and drop casting used for their preparation [10,11,12]. Of these methods, a cost-effective, simple, and reproducible method worth examining is electrospinning.

Electrospinning is a highly customizable technique that is used to create polymeric fibres in diameters typically ranging from 100–1000 nm [13]. Owing to the high surface area of these electrospun films, they can be used for different applications such as textiles [14], composites [15], filters [16], tissue engineering [17], and drug delivery [17]. Various polymers can be employed, and therefore, a range of properties, including hydrophobicity, porosity, conductivity, transparency, etc., can be tailored. The high surface area of the nanofibres permits the extensive and controlled incorporation of other photoactive materials. For example, the porous nature of some films can allow for the inclusion and delivery of smaller nanoparticles [18], molecules [19], or even gasses [20] after a photo-driven process. The incorporation of these nanomaterials in electrospun films is possible before or after the electrospinning process through a variety of methods that will be discussed (vide infra). As such, electrospun nanofibres are excellent materials for complex charge and energy transfer processes.

This review discusses the process of electrospinning nanofibres and the various energy and charge transfer systems with nanofibre films that have been reported to date. For this review, the participating additives in these processes will be limited to molecules, quantum dots, and metal nanoparticles. The charge and energy transfer processes discussed include Förster resonance energy transfer (FRET), photoinduced electron transfer (PET), metal-enhanced fluorescence (MEF), and upconversion. The current applications of these films are also discussed.

## 2. Electrospinning: How It Works

**Figure 1:**
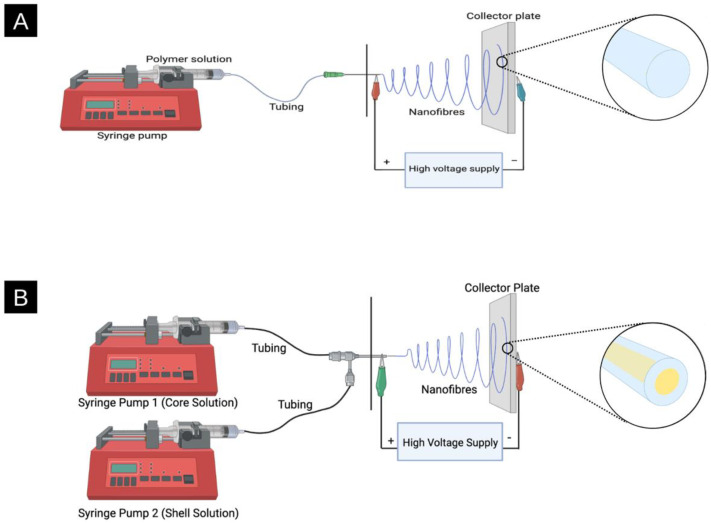
(**A**) Monoaxial electrospinning system where a polymeric solution is placed in a syringe pump and through a specific feed rate, is fed through tubing towards the metal spinneret. An electric field is produced by connecting the spinneret and collector plate to a power supply, which results in fibre formation (vide infra). A graphic representation of a monoaxial fibre is shown coming out of the collector plate. (**B**) A coaxial (core@shell) electrospinning system displays two syringe pumps containing the core solution and the shell solution, respectively. The two solutions travel through the plastic tubing into the coaxial needle. A strong electrostatic force is applied, and a Taylor cone forms a jet, displacing the nanofibres onto the collector plate. A graphical representation of a coaxial fibre is shown coming out of the collector plate, where the core is shown in yellow, and the shell is in blue.

Electrospinning converts polymeric solutions into nanofibre films. The solution typically consists of an organic solvent or water to dissolve one or more polymers [21]. A general electrospinning setup can be found in Figure 1, consisting of a syringe and syringe pump, tubing, metal spinneret, power supply, and a collector. More specifically, a syringe that houses the polymeric solution is placed in a syringe pump. When the syringe pump is turned on, plastic tubing carries the solution towards a metal spinneret. A high voltage supply is connected to both the spinneret and collector plate, and when turned on, a large potential (electric field) is applied between the two. The application of the electric field to the polymer solution that is being fed through the spinneret leads to fibre formation [13].

**Figure 2:**
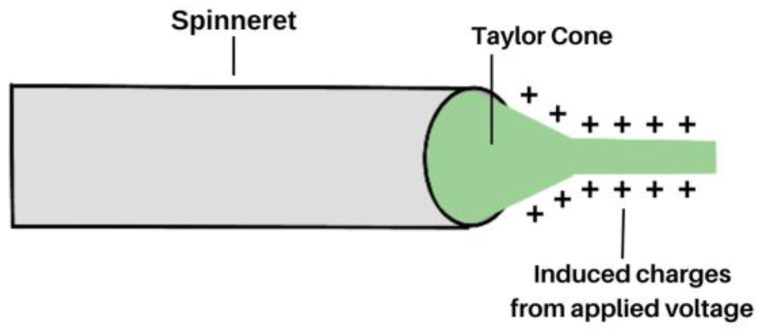
Representation of the Taylor cone formed at the tip of a metal spinneret when voltage is applied to a polymeric solution in the process of electrospinning.

Without the presence of a power supply, when a solution is pumped through the spinneret, it will form droplets owing to surface tension [22]. However, under a high voltage, surface charges become uniform (typically positive). Charge repulsion overcomes the surface tension of the droplet to form a Taylor cone and ultimately a jet, as shown in Figure 2. Between the spinneret tip and collector plate, the jet experiences instability, resulting in a circular lasso or whipping motion. The nanofibre jet’s diameter is affected by various parameters such as rate of solvent, evaporation, and charge density, vide infra [23,24,25,26,27].

**Figure 3:**
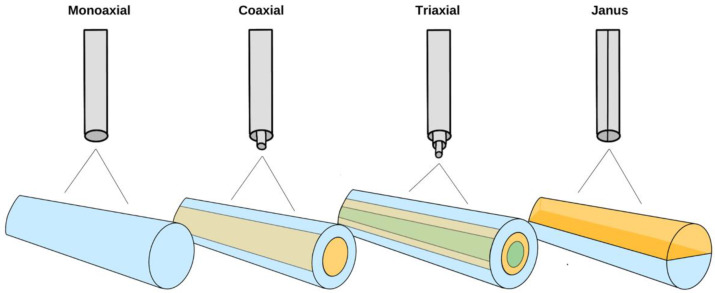
The different types of fibres that can be fabricated via electrospinning, including monoaxial, coaxial, triaxial, and Janus fibres. Monoaxial nanofibres are formed from a single solution of one polymer or a polymer blend, resulting in homogenous fibres. Coaxial nanofibres result from two separate solutions being fed through a separate core and shell component of a spinneret needle. Triaxial nanofibres result from three separate solutions being fed through a single spinneret. Janus fibres are formed by feeding two separate solutions through side-by-side spinnerets.

The metal spinneret used while electrospinning can be customized for unique fibre formation in terms of size (diameter) and structure (hierarchical). The spinneret is typically a cylindrical metal needle ranging from 4–30 Gauge [28]. Different sizes (diameter) of spinnerets can affect fibre diameter. For example, it has been previously observed that as the inner diameter of the spinneret decreases, the diameter of the fibre also decreases [29]. Monoaxial electrospinning is typically viewed as the traditional method of electrospinning, where a spinneret supports a single solution that can contain a single polymer or blend. Coaxial electrospinning allows for two separate solutions to be fed through a single spinneret, resulting in the formation of a core@shell structure, as shown in Figure 3 [30]. There are numerous advantages to coaxial electrospinning, such as the versatility in the choice of polymers, the confinement of various additives, and improving the spinnability of less soluble polymers [31,32]. As a result, coaxial electrospinning allows for greater photoactive additive stability, complete additive encapsulation, and tighter control for the release of agents while maintaining the functional activity of molecules [33,34]. Triaxial electrospinning involves an inner (core), middle (intermediate), and outer (shell) solution using three concentric needles attached to three different pumps to deliver the materials and create a multicoated fibre [30]. Finally, some spinnerets have side-by-side nozzles, resulting in Janus fibres (Figure 3).

**Figure 4:**
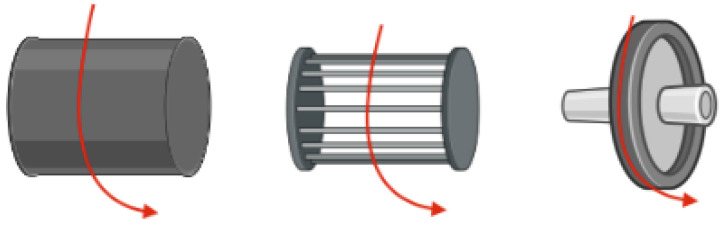
Three different types of rotating collectors can be used in electrospinning, including the drum (**left**), wired drum (**middle**), and disk (**right**). These rotating collectors allow for the formation of aligned fibrous films.

The orientation of the fibres that are displaced onto the collector can either be aligned or unaligned with one another, depending on the type of collector being used [35]. As previously discussed, unaligned fibres are randomly oriented on the collector plate, whereas aligned fibres (parallel with one another) can be achieved using rotating collectors, as shown in Figure 4. Aligned fibres can be collected using a rotating collector such as a rotating drum, wire drum, or rotating disk, where the speed at which a collector rotates can be set [36,37,38,39,40].

### 2.1. Factors That Affect Fibre Formation

The fibres obtained from electrospinning can vary greatly depending on the solution properties and processing parameters used. This includes the polymer’s molecular weight (MW), concentration and viscosity of the polymer in solution, the distance between the collector and spinneret, applied voltage, flow rate, solvent choice conductivity, flow rate, and environmental factors. Additional factors affecting fibre formation in electrospinning, which will not be discussed in greater detail in this review, include surface charge density in the polymer jet, surface tension, permittivity, etc. [41].

#### 2.1.1. Polymer Molecular Weight and Concentration

**Figure 5:**
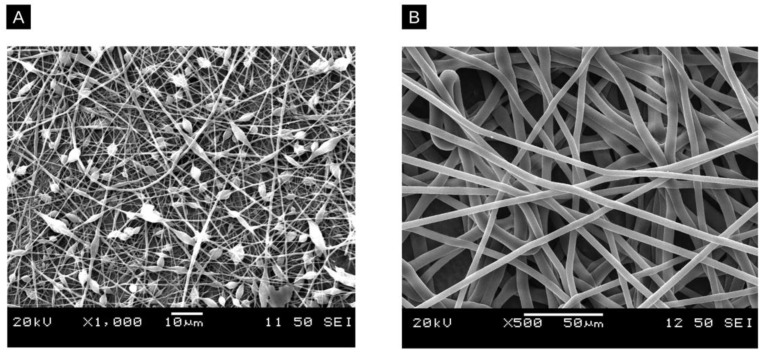
Comparative SEM images of a polystyrene (PS) nanofibre film prepared with two different concentrations. (**A**) shows a beaded film prepared with a lower concentration of 10% and (**B**) shows a film with a higher concentration of 20%.

The molecular weight (MW) and concentration of a polymer influence solution viscosity, surface tension, chain entanglements, and the morphology of resulting fibres [42]. If the molecular weight of a polymer is too low, viscoelastic forces decrease dramatically, and surface tension plays a larger role in the morphology of the fibres, resulting in beaded fibres [43]. An example of this is shown in Figure 5A, where two examples of polystyrene films are compared. Similarly, if the polymer solution is considerably dilute, the intermolecular distance is vast, resulting in weak intermolecular interactions, making it difficult to form fibres.

Generally, increasing the polymer MW and/or concentration up to a certain limit improves the morphology of the resulting fibres. Both parameters play a role in forming chain entanglements, which refers to the interlocking of polymer chains. Stable fibre formation occurs at >2.5 entanglements per chain, which can be met by increasing the concentration of the solution or MW of the polymer [44]. The concentration also affects the fibre diameter; as the solution concentration increases, the diameter typically increases as well [45].

When the polymer concentration and MW are too low or too high, fibre formation or morphology is adversely affected. Polymers with a MW that is too high have been observed to create flat and aggregated nanofibres with decreased homogeneity [46,47]. Similarly, the concentration of the solution must not be so high that its viscosity prevents the ability of the polymer to be stretched by the electric field [41]. For example, a study found that increasing the concentration of polylactic acid (PLA) increased the diameter of the resulting fibres [48]. Another study found that as the concentration and MW of polyethylene glycol (PEG) in solution increased, the resulting fibres had decreased beading and more consistent diameters [43].

#### 2.1.2. Viscosity

As alluded to above, solution viscosity plays a key role in fibre formation as it can both promote or impede fibre formation [49]. Optimal solution viscosity produces continuous fibre formation during the jet creation; however, if the viscosity is too high, the fibre jet may not be pulled effectively [25]. For example, one study found that when electrospinning poly(carbonate urethane) (PCU) with low, intermediate, and high viscosity the resulting fibres were beaded, uniform, and large in diameter, respectively [49]. In addition, when electrospinning coaxial nanofibres, the viscosity of the shell solution plays an important role in ensuring the formation of a core@shell structure [50]. For example, the effects of solution viscosity on the core@shell (polycaprolactone@polyethylene oxide, PCL@PEO) structure diameter and pore size have been examined. The results showed that the higher the viscosity, the larger the fibre diameter and pore size [51].

#### 2.1.3. Applied Voltage and Tip-to-Collector Distance

The tip-to-collector distance needs to be taken into consideration alongside the voltage, as the two play a role in fibre formation [52]. The distance between the tip and the collector is where the elongation of the fibres occurs and where the solvent evaporates. Thus, if this distance is too large, the fibres will not be able to reach the collector successfully. On the other hand, if the tip-to-collector distance is too short, the solvent cannot evaporate completely, resulting in the fibres lacking elongation [42]. Distance also affects the fibre diameter; generally, the greater the distance, the lower the diameter of the fibres [42]. However, changing the tip-to-collector distance cannot be done without considering the concentration and applied voltage as well. For example, one study found that at a higher concentration of starch, a higher voltage alongside a greater tip-to-collector distance was needed to produce consistent fibres. Considering the high viscosity of the polymeric solution, longer distances allowed for sufficient elongation of fibres from the whipping motion [52]. If a solution is prone to beading, a larger tip-to-collector distance typically results in larger beads [53].

**Figure 6:**
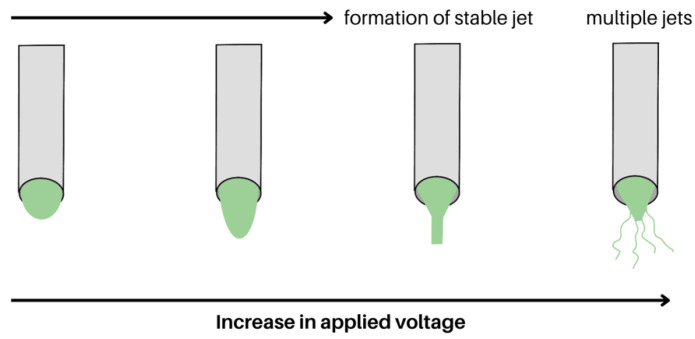
Schematic of a polymer solution at the tip of a spinneret, experiencing different voltages. As the applied voltage increases, a stable jet is formed that can allow for nanofibre formation (shown emerging from the third spinneret). When the applied voltage passes a certain point, the jet experiences more instability, and the formation of multiple jets will occur (shown emerging from the fourth spinneret).

Whether through increasing the applied voltage or decreasing the distance, the surface charge on the Taylor cone heavily influences the droplet shape that leads to the jet formation, as observed in Figure 6. As a result of the electric field, four different types of jets will be formed, namely (i) stable jet, (ii) fluctuating jet, (iii) stable jet with polymer drops, and (iv) multiple jets [54]. A stable jet occurs when the jet flow rate is lower than the feed rate, with enough solution emerging to form a stable jet. At higher applied voltages, the jet flow rate is higher than the feed rate, leading to a fluctuating jet that forms beaded fibres. This occurs because the jet flow rate of the polymeric solution is faster than the velocity at which the solution can be replenished at the needle tip, leading to a fluctuating jet [55,56]. A stable jet with polymer drops, on the other hand, will occur when the polymer solution builds up at the tip of the needle and drops after some time. In this case, the beads are more globular due to a lower applied voltage. Finally, if the applied voltage is high, multiple jets extrude at the same time from the tip of the needle, and fibres with elongated beads are produced [55].

The applied voltage can affect the morphology of the fibres. One study found that as the applied voltage increased, the surface of the fibres would be coarser (i.e., more bumps on the surface). This was due to the increase in the jet flow rate and the decreased travelling time of the jet, which prevents fibres from being sufficiently stretched [15]. The effect of the applied voltage typically follows a pattern in which increasing the voltage decreases the fibre diameter, as there is an increase in electrostatic forces acting on the jet resulting in thinner fibres [57,58,59]. In another study, increasing the applied voltage on poly(vinylidene difluoride) (PVDF) resulted in an increase in diameter with a broad distribution, but further increasing the voltage decreased the diameter and resulted in more uniform fibres [54]. Examples in which the fibre diameter increased as the voltage increased usually occur due to an increase in the solution concentration [60,61]. However, there are instances when as the voltage increases, so does the fibre diameter, without a change in the concentration. In one example, this was also accompanied by a broader size distribution [62].

#### 2.1.4. Conductivity

Owing to the role of surface charges in fibre formation, solution conductivity plays a large role in electrospinning. Higher conductivity can allow for the enhanced elongation of a jet, resulting in thinner or thicker fibres. Thicker fibres can result from increased mass flow due to noncovalent interactions between additives and the polymer [63]. Nonetheless, improving the solution conductivity can help increase the spinnability of certain polymer solutions [30]. One study found that improving the conductivity also improved the spinnability of the solution through the addition of a salt (ammonium salt, TEAB) to a solution of polymers of intrinsic microporosity (PIMs) in 1,1,2,2-tetrachloroethane (TeCA). In this case, the inclusion of TEAB did increase the mean fibre diameter [64]. However, cases in which increasing conductivity resulted in thinner fibres have been found in a variety of polymers as well, including polystyrene (PS) [65], polyethylene oxide (PEO) [66], and poly(L-Lactic acid) (PLA) [67].

#### 2.1.5. Flow Rate

**Figure 7:**
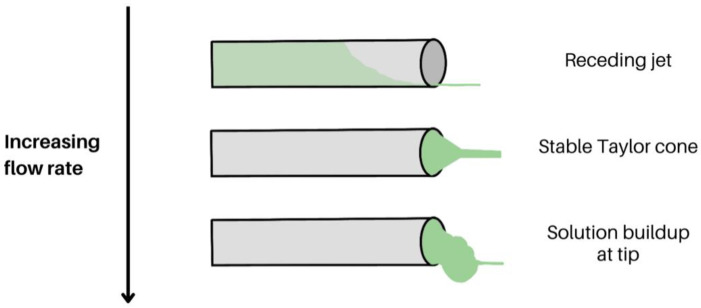
Taylor cone and jet stability with regards to increasing flow rate from top to bottom: receding jet, stable Taylor cone, and solution build-up at the tip of the spinneret.

Closely related to the applied voltage, the flow rate also has an influence on the formation and morphology of electrospun nanofibres as observed in Figure 7. Several studies have shown that increasing the flow rate increases the diameter and size distribution of fibres, but if the flow rate is too high, it results in the formation of beads on a string with large diameters or even nanoparticles with no fibres [68,69]. This is due to the flow rate exceeding a critical value, where the delivery rate of the fluid to the tip of the spinneret is higher than the rate at which the solution is being pulled by the electric field [68,69]. Conversely, a flow rate below a critical value would result in an unstable Taylor cone, as the solution would recede into the needle and the cone would disappear. The Taylor cone would eventually replenish as the available solution would be pulled, but this would result in a large size distribution of the fibres [68].

The flow rate also influences the porosity of the resulting fibres. It has been demonstrated that increasing the flow rate decreases the porosity of polyvinyl alcohol (PVA), Nylon-6, and polyvinyl pyrrolidone (PVP) fibres. This is most likely due to the increased fibre diameter and density as more polymer is released from the spinneret at a faster rate [70].

#### 2.1.6. Solvent

There are other factors that also need to be considered related to the formation of fibres. This includes the solvent that is used, as each one will have a different surface tension and boiling point, which will require the application of a different voltage. Solutions with high surface tension may be difficult to electrospin, and as a result, beads on a string could form, as demonstrated by Yang et al. and Chuangchote et al. [71,72]. In other studies, authors electrospun PVP in various solvents, using a constant concentration to observe the effect of the solvent on resulting fibres. The authors were able to conclude that water, dimethylformamide (DMF), and dichloromethane (DCM) led to beaded fibres, while solvents with lower surface tension (i.e., methanol and ethanol) produced non-beaded fibres [73,74].

#### 2.1.7. Environmental Factors

**Figure 8:**
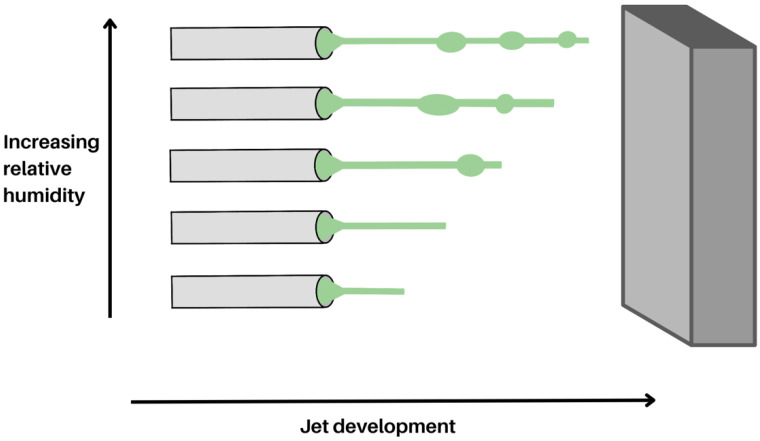
Jet solidification at various relative humidity levels, leading to different fibre morphology. As the relative humidity increases, so does the possibility of creating fibres with beads on a string.

While these can be difficult to control, environmental factors such as temperature and humidity also need to be considered. Increasing the relative humidity (RH) can result in decreased fibre diameter, including the formation of beads or no fibres, as demonstrated by several studies (Figure 8) [75,76,77,78]. When RH decreases, the solvent rate of evaporation increases, resulting in the solidification of the jet occurring earlier. However, when RH is higher, the rate of evaporation is lower, and solidification happens later in the process, so the jet is exposed to the applied voltage for a longer time, resulting in the stretching and thinning of the jet, leading to fibres with smaller diameters. Beads could also form if the relative humidity is too high owing to a lower solvent evaporation rate. This results in the elastic forces eventually overcoming the plastic forces, creating beads [75].

Temperature has an influence on the viscosity of the solution, which affects the spinnability. It was demonstrated that the viscosity decreased when the temperature increased, as it is governed by an Arrhenius-type activation energy (Equation (1)) [79,80].

η = Aexp (E_a_RT) (1)

where η is the viscosity, A is the pre-exponential factor, E_a_ is the activation energy of the flowing solution, R is the gas constant, and T is the temperature of the solution [80]. Temperature also influences the morphology of the fibres, as it was seen that fibre diameter decreases as the temperature increases due to a decrease in surface tension and viscosity [79].

### 2.2. Light-Harvesting Materials in Electrospun Nanofibres

Considering the parameters listed above that affect electrospun nanofibre morphology, the addition of light-harvesting materials in electrospun films can also affect the required electrospinning parameters [81]. Herein, the various methods of incorporating light-harvesting materials in electrospun nanofibres and the experimental conditions used in their preparation are elaborated. It should be noted that values such as applied voltage vary between instruments owing to inherent differences in the equipment and are therefore excluded.

**Figure 9:**
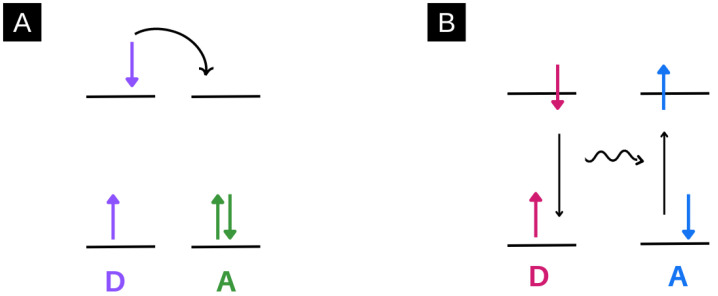
An example of a charge transfer process (**A**) and energy transfer process (**B**). (**A**) describes a charge transfer process whereby an excited electron from a donor is transferred to an acceptor. (**B**) describes an energy transfer process whereby an excited electron in a donor relaxes back down, and the resultant energy is transferred to an acceptor. This results in a ground-state electron in the acceptor being excited.

This review discusses energy and charge transfer processes within electrospun nanofibres, which can exist between various nanomaterials such as nanoparticles, quantum dots, and molecules. While the specific energy or charge transfer systems will be discussed in more detail below, the difference between energy and charge transfer is elaborated here in Figure 9. In this review, the transfer of an electron after a photo-driven process is described when referring to charge transfer systems. In such processes, an electron is topically excited by light and transferred through a variety of processes, vide infra [82].

In terms of energy transfer systems, this process refers to the transfer of energy after excitation by light. This typically occurs between a donor and acceptor after a photo-driven process. Typically, in these processes, an electron is excited, and instead of the electron being transferred, it relaxes back down, and energy is transferred instead, vide infra [83].

Both charge transfer and energy transfer systems have a variety of applications, especially within electrospun nanofibres. For example, these applications include sensing, photovoltaic devices, and luminescent solar concentrations, vide infra.

## 3. Energy Transfer Systems within Electrospun Nanofibres

Owing to the versatility of electrospun nanofibres and the variety of parameters that can be altered to improve spinnability, the development of films from polymeric solutions using electrospinning is advantageous. Perhaps most unique is the ability for various energy and charge transfer processes to occur in these films, making them highly photoresponsive. Energy transfer systems such as Förster resonance energy transfer (FRET), metal-enhanced fluorescence (MEF), and upconversion and downconversion can occur between molecules, nanoparticles, metals, and between like-species [83,84]. These processes typically turn fluorescence on or off or enhance emission. In addition, they are often highly sensitive to their environment, have a rapid response rate, and have a low detection limit, leading to a variety of applications [85,86]. Critical to realizing these applications is an effective matrix to house the energy-transferring and/or accepting species. Owing to their large surface area, high tunability, and ease of fabrication, electrospun nanofibres are an excellent structural matrix for light absorption and energy transfer.

**Figure 10:**
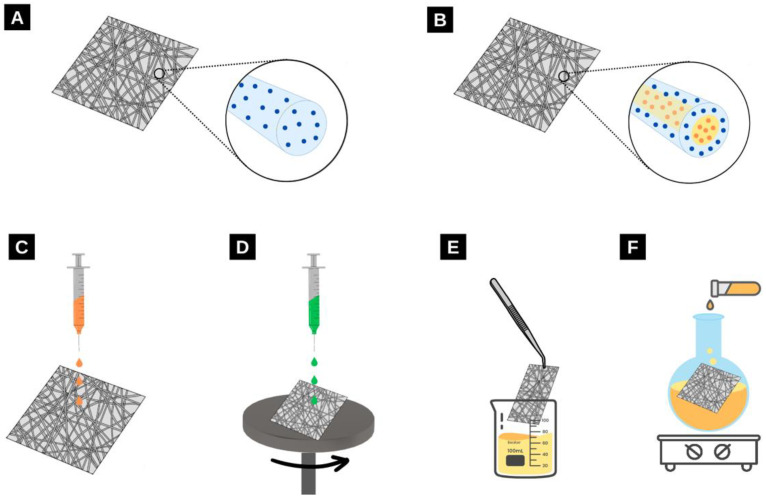
Various methods that can be used to incorporate additives into electrospun nanofibres. (**A**,**B**) refer to the incorporation of additives in solution with polymers that are then electrospun as either (**A**) monoaxial or (**B**) coaxial nanofibre films. (**C**) Drop casting can be used to apply a layer of additives to electrospun films. (**D**) Spin-coating can be used to apply a thin layer of additive to electrospun films. (**E**) Films can be dipped into a solution and additives can be absorbed onto the fibres. (**F**) Various chemical post-processing methods can be employed such as dissolving the films in solution and growing nanoparticles on the surface of the fibres.

As alluded to previously, a myriad of materials can be incorporated within (and upon) electrospun nanofibres. For example, additives can be embedded in the fibres during the electrospinning process [87]. This involves preparing the solution with both the polymer and nanomaterial dissolved and spinning the prepared solutions into fibres. Another method includes coaxial electrospinning, where the light-harvesting species can be in either the core, the shell, or both. Finally, post-processing methods can be used where the nanomaterials can be added to the surface of electrospun nanofibres by drop-casting [88], spin coating [89], submerging the films in a solution [2], or via chemical post-modification, as shown in Figure 10 [90].

### 3.1. Förster Resonance Energy Transfer (FRET) in Electrospun Nanofibres

**Figure 11:**
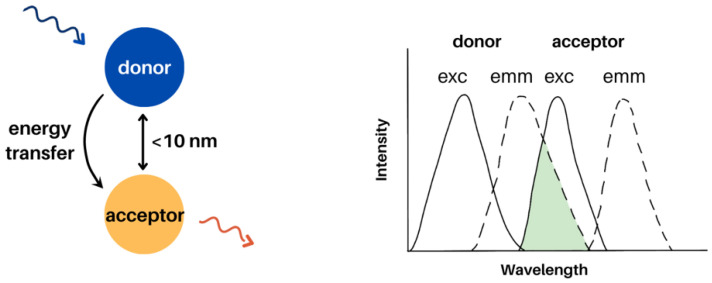
FRET requires spectral overlap between a donor emission and acceptor absorbance. The donor and acceptor must also be in close proximity, as FRET occurs at a distance of 10 nm or less.

Forster resonance energy transfer is a distance-dependent dipole-dipole interaction between donor and acceptor. FRET occurs at distances between 1–10 nm and requires spectral overlap between the donor emission and acceptor absorbance (Figure 11). Other factors that play a role in FRET include the quantum yield of the donor and the relative orientation of the donor and acceptor [91]. Examples of FRET donors and acceptors include molecules, fluorophores, and quantum dots. FRET processes using molecules and quantum dots in electrospun films are discussed herein.

#### 3.1.1. Molecules

**Figure 12:**
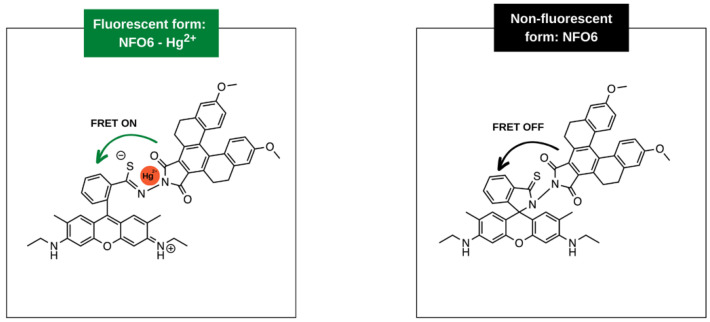
A molecular-based FRET system where NF06 was electrospun in polymeric films for the detection of mercury. When mercury is not present, the fluorophore is in a ring-close state preventing fluorescence. Upon introduction of mercury, the fluorophore moves to a ring open state, allowing intra-molecular FRET to occur after the donor is excited. Graphic adapted from [93].

Molecular-based FRET detection systems have been of great interest to researchers owing to their high sensitivity, low cost, tunability, and breadth of options [92]. Surprisingly, when FRET is paired with the ease of embedding molecules in electrospun nanofibres, only a handful of literature examples have been reported on electrospinning FRET-exhibiting molecules. Tonsomboon et al. electrospun a solution of turn-on FRET-based dyes in polycaprolactone (PCL) and cellulose acetate (CA) for the detection of mercury (II) [93]. The detection of mercury typically requires extra solution processing, increasing the time and cost. Embedding the sensing materials (donor and acceptor covalently bound to each other) in electrospun nanofibre films is more cost-effective, responsive, and highly sensitive, and the films can be easily extracted from contaminated samples. The authors designed a dye, NF06, by coupling to common dyes with a large Stokes shift (a helicene coupled with a rhodamine 6 G thiohydrazide). The nanofibre films were converted to test strips that would be submerged into various aqueous samples. Upon the binding of Hg^2+^, the molecule transforms from a ring-closed to a ring-open form, allowing for fluorescence, as shown in Figure 12. To obtain the electrospun films, a 7.5 wt% polymer blend of cellulose acetate (CA) and polylactic acid (PLA) (1:1 wt/wt) in acetone and DCM (3:1 v/v) was used. The solution was fed through an 18 G needle at a rate of 5 mL/h, under a voltage of 18 kV and at a tip-to-collector distance of 10 cm. After electrospinning, the NF06 molecules were deposited on films via drop-casting (Figure 10C) and/or dip-coating (Figure 10E). The strips began exhibiting fluorescence at a mercury concentration of 10 ppb and plateaued at 10 ppm, exhibiting high sensitivity and saturation, respectively [93].

**Figure 13:**
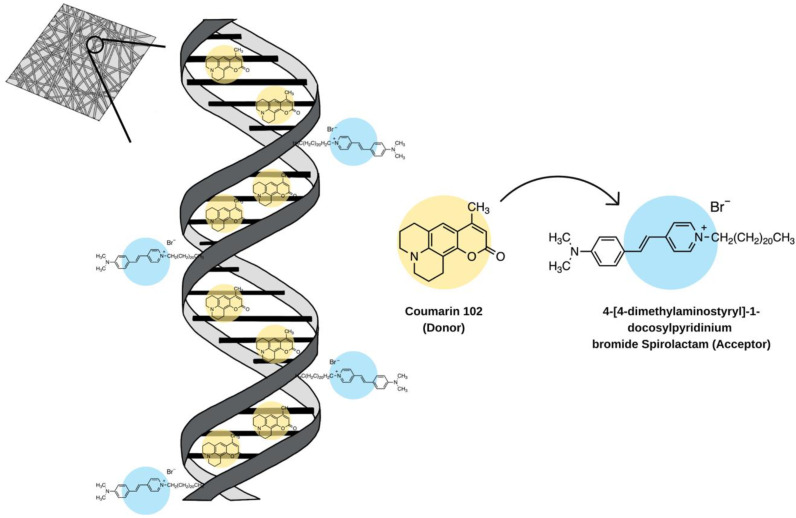
A FRET donor (Coumarin 102) and acceptor (4-[4-dimethylaminostyryl]-1-docosylpyridinium bromide Spirolactam) incorporated in salmon DNA and electrospun into nanofibre films. The donor localizes between the base pairs and the acceptor localizes to the minor groove of the backbone. CTMA, a surfactant, was used but not shown in this graphic for clarity. Molecules are not shown to scale [87].

FRET-based systems have also been reported for light-emitting applications in electrospun nanofibres [87]. A study by Ner et al. incorporated a donor and acceptor pair conjugated to salmon DNA (500 kDa) [87]. The use of DNA in optoelectronic devices is of interest because of its thermal stability, highly organized structure, and transparency. By using two dyes that have unique interactions with DNA, the donor and acceptor were kept at specific distances from one another (Figure 13). The authors explored different ratios of donor to acceptor, which showed that a molar ratio of 1:20 resulted in the film appearing as pure white. To obtain the electrospun films, a 10% (wt/wt) solution of DNA-CTMA (cetyltrimethylammonium chloride) in ethanol/chloroform (3:1 v/v) was prepared. The solution was fed through at a rate of 0.8 mL/h, a tip-to-collector distance of 17 cm, and a voltage of 20 kV [87].

**Figure 14:**
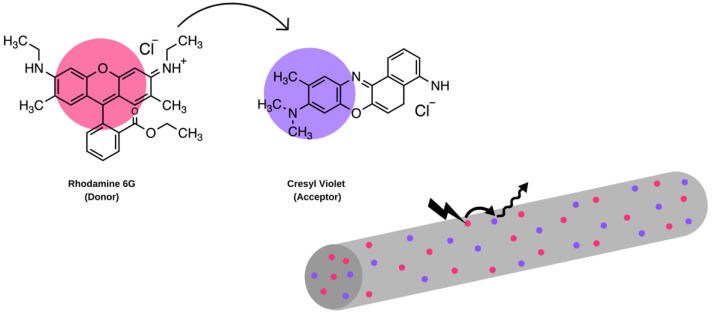
FRET donor (rhodamine 6 G) and acceptor (cresyl violet) incorporated into monoaxial nanofibres that exhibit FRET [19].

FRET-based systems can also be used for lasing applications in display and sensing technologies. For such applications, embedding the FRET-exhibiting dyes in electrospun nanofibres is an efficient method of producing lasing-compatible films. Sznitko et al. incorporated rhodamine 6 G (donor) and cresyl violet (acceptor) into PMMA (Figure 14). Various concentrations of both dyes dissolved in a 30% PMMA in chloroform and DMF (4:1 v/v) were reported. The solution was fed at a rate of 0.7 mL/h, a tip-to-collector distance of 25 cm, and a voltage of 18 kV. The authors also measured the FRET efficiency and found it to be 57% at a 1:1 donor-to-acceptor concentration. Changing the concentrations by increasing the acceptor would decrease the FRET efficiency to 49% [19]. Kaerkitcha et al. also explored FRET between molecules in electrospun nanofibres where pyrene and porphyrin-based dyes were incorporated in hyaluronic acid [94].

**Figure 15:**
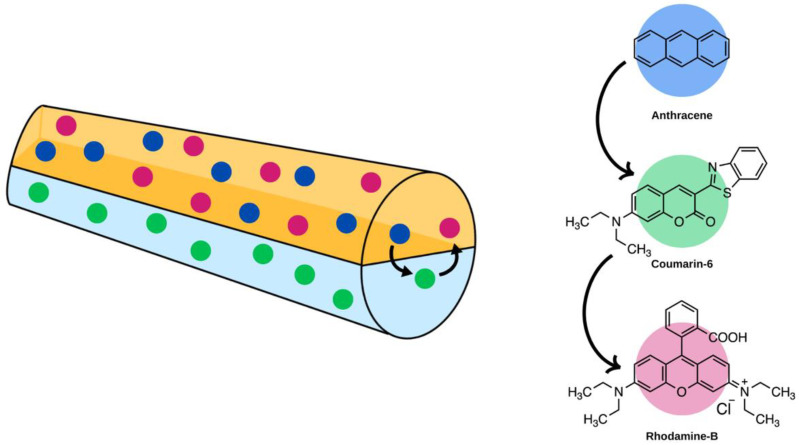
Janus electrospun nanofibres that have a two-step FRET system incorporated. The fluorophores incorporated within included anthracene and rhodamine-B in the PVP layer and coumarin-6 in the PAN layer. Upon excitation, anthracene transferred energy to coumarin-6, which then transfers to rhodamine-B [95].

Two-step energy transfer systems can also be explored in electrospun nanofibres, as examined by Qin et al. using a Janus structure to electrospin polyvinylpyrrolidone/polyacrylonitrile (PVP/PAN) fibres. In this study, two molecules (anthracene and rhodamine-B) were incorporated in PVP and one (coumarin-6) in PAN. The energy transfer system occurred from anthracene to coumarin-6 and then to rhodamine-B, as shown in Figure 15. By adjusting the concentrations of the various molecules, the authors were able to create white-light-emitting nanofibres. These films were electrospun using a 21 G needle tip, a flow rate of 0.003 mL/min, a tip-to-collector distance of 15 cm, and a voltage of 15 kV [95]. Another two-step energy transfer process was explored by Vohra et al. where the host polymer blend, perfluorotributylamine (PFTBA) and polyethylene oxide (PEO), acted as the donor, and zeolite L crystals functionalized with various fluorescent molecules acted as the acceptor [96].

**Figure 16:**
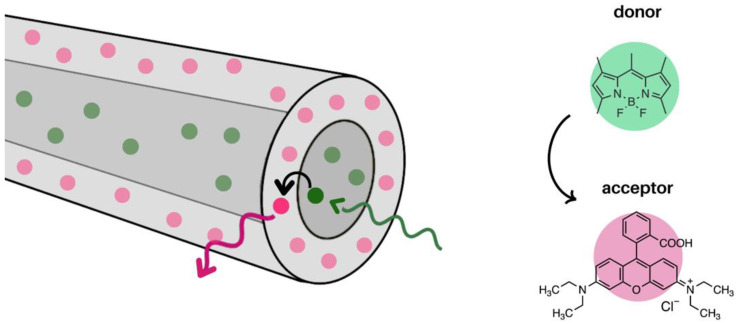
FRET from core to shell in a coaxial nanofibre film. BODIPY was embedded in the core and rhodamine-B was embedded in the shell. FRET occurred from core to shell [97].

The aforementioned examples of FRET within electrospun nanofibres are limited to monoaxial films. However, FRET within coaxial nanofibres has also been explored. Mahmood et al. demonstrated an energy transfer process from donors in the core to acceptors in the shell of a core@shell nanofibre. Specifically, BODIPY was incorporated in polyvinyl pyrrolidone (PVP) as the core, and rhodamine-B was incorporated in PVP as the shell, Figure 16 [97]. In this study, using a core@shell structure allowed for the controlled localization of the donor and acceptor as well as an increased surface area between them. The films were electrospun at a flow rate of 1.0 mL/h, 23 kV, and a 17 cm tip-to-collector distance. Upon excitation of the BODIPY, the emission of rhodamine was observed. The FRET efficiency of the system was 54% [97].

#### 3.1.2. Quantum Dots

**Figure 17:**
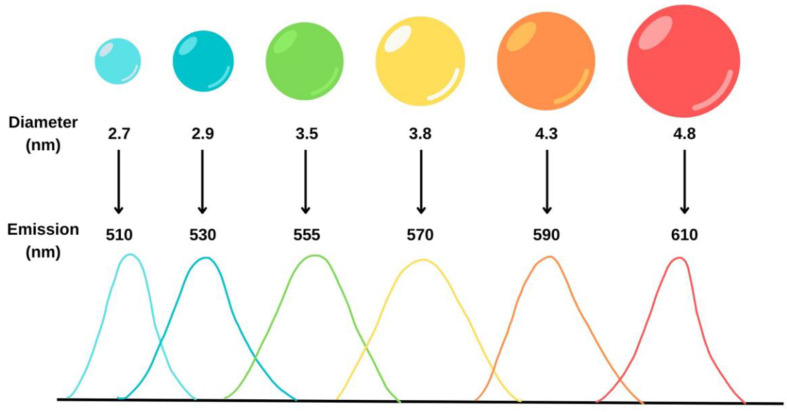
Emission of quantum dots can be changed by tuning the nanoparticle size. As the size increases, the emission wavelength redshifts [101].

Quantum dots (QD) are semiconductor nanocrystals comprised of around 100–100,000 atoms, resulting in an overall size of 2–10 nm [98,99]. QD are typically composed of groups II–VI and III–V elements, such as Cd, Hg, Se, Pb, Zn, etc. [99]. Quantum dots emit fluorescence owing to electrons relaxing from the conduction band to the valence band. By tuning the particle size of these materials, various emission wavelengths can be observed as shown in Figure 17 [100,101]. Quantum dots exhibit high quantum yields and resistance to photobleaching [102].

The application of quantum dots as donors or acceptors in FRET-based systems has been widely studied [103]. Similar to molecules, for FRET to occur between quantum dot pairs, there must be appropriate spectral overlap and distance between the two species [101,103]. However, quantum dots offer many advantages compared to molecules, including a greater FRET distance (can go beyond 10 nm) and higher extinction coefficients (10^6^ M^−1^cm^−1^ for quantum dots compared to 10^−5^ M^−1^cm^−1^ for organic dyes) [103]. Owing to these advantages, quantum dots have been embedded within electrospun nanofibres as FRET pairs.

In one example, Altintas et al. loaded green- and red-emitting quantum dots into nanofibres and observed energy transfer between the pairs at varying concentrations [104]. The quantum dots were loaded into polycaprolactone (PCL) (0.096 g/mL) in a solvent system consisting of THF and DMF (4:1 v/v). The solutions were then fed through an 18 G spinneret, at a voltage of 15 kV, a feed rate of 1–1.5 mL/h, and a tip-to-collector distance of 25 cm. To observe energy transfer, the authors used time-resolved photoluminescence (PL) spectroscopy. Upon introduction of the acceptor red quantum dots, donor lifetime decreased, indicating that FRET was occurring. The FRET efficiency changed depending on the concentration of donor-to-acceptor as well as the distance between the two [104].

**Figure 18:**
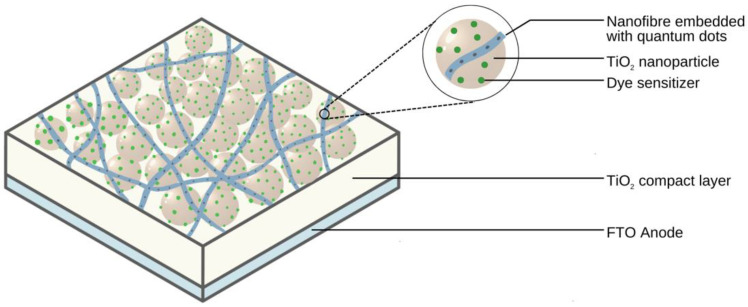
A graphical representation of incorporating nanofibre embedded with quantum dots in the anode of a dye-sensitized solar cell (DSSC). In this example, a poly(methyl methacrylate) (PMMA) nanofibre film doped with CdSe quantum dots was deposited on a TiO_2_ mat. Adapted from [105].

Another study by Choi et al. assessed the spatial distribution of quantum dots within nanofibres and its effect on FRET [105]. Quantum dots can act as FRET donor layers in dye-sensitized solar cells (DSSCs). Electrospinning these nanomaterials into fibres allows for the easy incorporation of these as a layer in the DSSC, preventing direct contact of the quantum dots with other components and controlling the spatial distribution of each quantum dot to prevent self-quenching, as shown in Figure 18. The CdSe quantum dots were dissolved in chloroform and poly(methyl methacrylate) (PMMA) (0.1–0.2 g/mL). The solution was electrospun at a flow rate of ~20 μL/m, a voltage of 13 kV, and 10 cm between the spinneret and collector plate. The results showed that the spatial distance between quantum dots can be controlled by altering the diameter of the nanofibre. The authors observed that the narrower the fibre, the greater the distance between quantum dots. Upon incorporation of the films into DSSCs, a 25% enhancement in the performance of the DSSC was reported [105].

In some cases, quantum dots have been used solely as the donor in nanofibres for sensing applications. For example, CsPbBr_3_ perovskite quantum dots (CPBQD) were embedded in PMMA for sensing in an aqueous medium [106]. CPBQD films acted as a sensor for the detection of metal ions, proteins, and organic molecules [106]. In another study, CdSe@ZnS quantum dots were immobilized on the surface of PCL@TMSPEDA nanofibres and used to create a FRET-based nanofibre film sensor [107]. Drug release in electrospun films can also be monitored using FRET. In a study, the quenching of quantum dots was decreased upon drug release, as there was decreased FRET between the quantum dots and the drug [108].

### 3.2. Metal-Enhanced Fluorescence

**Figure 19:**
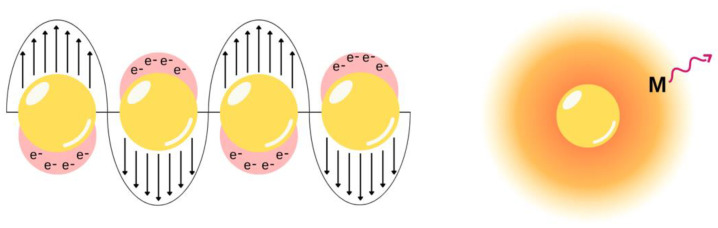
Metal-enhanced fluorescence occurs owing to surface plasmons generated from a metal nanoparticle interacting with incident light. Surface plasmons refer to the collective oscillation of surface electrons as shown on the left. The metal nanoparticle can cause enhanced emission of a nearby fluorophore through a variety of pathways. One example is shown on the right, whereby a molecule (M) is within the generated electromagnetic field of the nanoparticle.

Metal-enhanced fluorescence (MEF) is a process whereby a fluorophore in certain proximity to a metal nanostructure can exhibit amplified fluorescence (Figure 19) [109]. This is often quite useful for sensing applications to improve detection or for photovoltaic devices to improve performance. Metal nanostructures can interact with incident light in many notable ways, some of which include light scattering, enhancing the local electromagnetic field, and non-radiatively transferring energy. When a fluorophore is in close proximity (typically within 5–90 nm) with a metal nanostructure and there is spectral overlap between the metal and fluorophore, then this can result in enhanced fluorescence of the fluorophore [109]. These interactions can occur within spin-coated films with fluorophores on a metal-coated surface [110] and in bilayer vesicles in an aqueous medium [111]. Alternatively, electrospun nanofibres offer an excellent matrix for MEF studies owing to their versatility in design including choice in polymer, hierarchical design, control over the distance between nanostructures, and more. Owing to these benefits, the design and use of electrospun films with MEF abilities has been explored.

**Figure 20:**
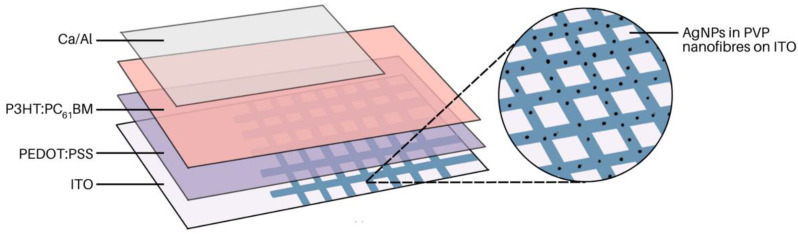
AgNPs embedded in polyvinyl pyrrolidone (PVP) nanofibres coated on ITO. This film was used as an additional light-harvesting layer in an organic photovoltaic device. Adapted from [112].

MEF has been used in a variety of studies to enhance the performance of solar cells. For example, in organic photovoltaic (OPV) devices, a thin light-harvesting layer is preferred to prevent charge recombination. However, this lowers the light-harvesting ability of the device; conversely, light trapping via MEF helps enhance OPV device performance. Chen et al. explored this by fabricating electrospun nanofibres with AgNP@PVP and incorporating this as a layer within OPV devices (Figure 20) [112]. A core@shell structure was fabricated with the AgNP in the core and PVP in the shell. The solutions were electrospun at a feed rate of 0.1 mL/h, a voltage of 14–15 kV, and a tip-to-collector distance of 13 cm. The fibre mat was placed between the indium-tin-oxide (ITO) substrate and PEDOT:PSS layer. Upon incorporation of the electrospun film, device performance was 4.19%, compared to 3.53% without the additional light-harvesting film. Nanofibre films with MEF characteristics have also been used to develop other devices. For example, the effect of AgNPs on the development of white-light-emitting diodes has been explored [113]. In another study, dual-function films were developed to improve OPV efficiencies. The films consisted of luminescent solar concentrating (LSC) nanoparticles and AgNPs to achieve a dual-functioning film [18].

Camposeo et al. explored the influence of AuNP on the emission of rhodamine when electrospun in PVP. PVP was chosen owing to its optical transparency in the visible spectrum. Rhodamine exhibits excitation and emission at 540 and 565 nm, respectively, which matches the optical features of the AuNPs (60 nm in size) that have an excitation spectrum peaking at 538 nm. To prepare the solution for electrospinning, PVP and rhodamine were dissolved in a solution of ethanol and AuNP in water. The weight ratio prepared was 1:10 rhodamine to PVP and 1:100 AuNP to PVP. The samples were electrospun at a flow rate of 0.5 mL/h, a tip-to-collector distance of 15 cm, and a voltage of 11 kV. Similarly, in another study by Burris and Cheng, the effect of AgNPs on the fluorescence on the polymer polyethylene oxide/polydiacetylene (PEO/PDA) blend was explored. Various sizes of AgNPs were incorporated in PEO/PDA electrospun nanofibres and a 4.6-fold enhancement of fluorescence was observed [114].

Metal-enhanced fluorescence is often exploited to improve sensor activity. Yun et al. designed a biosensor by electrospinning polycaprolactone (PCL) films decorated with silica-coated AgNPs for antibody detection. A 20% solution of PCL in TFE was prepared and electrospun using a flow rate of 0.6 mL/h, a voltage of 10 kV, and a tip-to-collector distance of 20 cm. The films were immersed in a solution of AgNO_3_ and exposed to UV light to promote the growth of AgNPs on the nanofibres. Following this, a silica layer was coated on the film. The film was used to sense fluorescently labelled antibodies, which exhibited enhanced fluorescence owing to the AgNPs [115]. In another study, Fan et al. developed a MEF-based sensor using AgNPs/SiO_2_ in polyacrylonitrile (PAN) [113].

### 3.3. Upconversion

**Figure 21:**
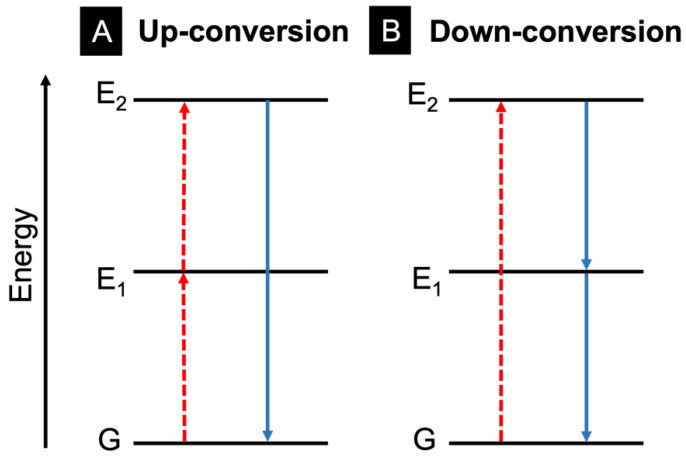
Upconversion (**A**) and downconversion (**B**) of electrons, visualized from ground-state energy level, G, to excited state energy level E_2_. The dashed (red) lines represent the initial electron excitation(s) while the solid (blue) lines depict electron relaxation and emission in each case.

Light can be upconverted or downconverted for use in various applications; upconversion converts multiple lower-energy wavelengths of light into a higher-energy photon, and downconversion converts a higher-energy photon into lower-energy wavelengths of light, as shown in Figure 21 [84]. This can occur within molecules and inorganic materials (particularly rare-earth metals). This review will focus on upconversion within electrospun nanofibres.

**Figure 22:**
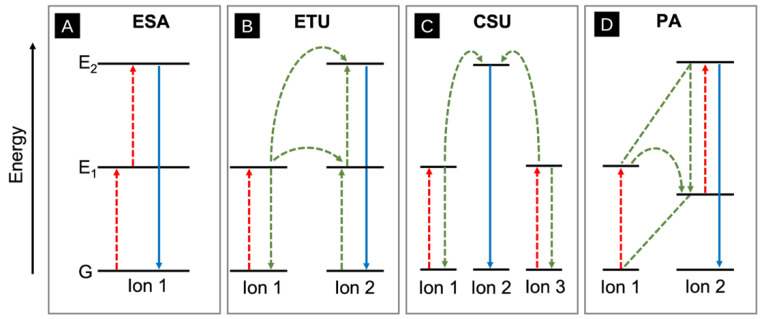
Upconversion processes. (**A**) Excited state emission (ESA), (**B**) Energy-transfer upconversion (ETU), (**C**) Cooperative sensitization upconversion (CSU), and (**D**) Photon avalanche (PA) upconversion. The red dashed lines represent electron excitation, the green dashed lines represent energy transfer, and the blue solid line represents electron relaxation and emission.

There are several mechanisms for the upconversion of light. One of these mechanisms is excited state absorption (ESA), which is the basic conversion pathway that involves the excitation of an electron from the ground state into the second excited state using a two-step excitation mechanism (Figure 22A) [116]. Another mechanism of upconversion is energy-transfer upconversion (ETU), which is outlined in Figure 22B. In this process, a similar two-photon absorption mechanism occurs but with two neighbouring ionic species [116]. First, a photon is absorbed by each ion to populate the E_1_ excited states. Next, one of these electrons relaxes down to the ground state using non-radiative energy transfer, while the other electron is promoted to the E_2_ excited state, resulting in light upconversion. Cooperative sensitization upconversion (CSU) involves two-photon absorptions and three adjacent atoms. The two outer atoms absorb a photon and simultaneously transfer their excitation energy to an acceptor atom, resulting in an excitation energy that is twice as large (Figure 22C) [117]. This energy-transfer process is commonly seen in lanthanide species such as Er^3+^, Tm^3+^, and Tb^3+^ [117]. Finally, photon avalanche (PA) upconversion occurs when an electron is excited in ion 1 to an energy state E_1_, and then electron transfer to ion 2 occurs. Consequently, this electron is promoted to a second excited state E_2_ within ion 2, after absorption of another photon (Figure 22C) [118]. PA differs in that the E_1_ values for ion 1 and ion 2 in PA are not degenerate. Sufficient overlap of the participating energy levels in ions 1 and 2 is necessary for this process to occur.

**Figure 23:**
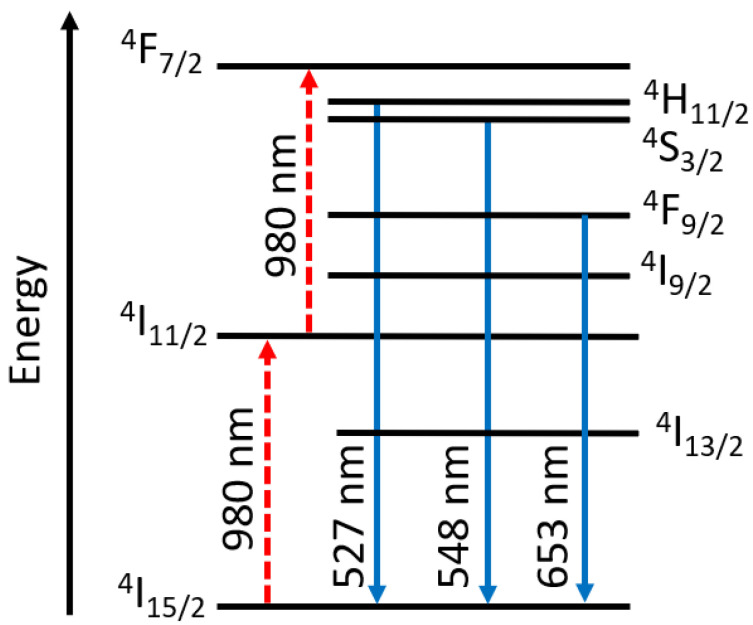
Upconversion process within the 4f energy levels of Er^3+^ using a 980 nm excitation wavelength. Adapted from [120].

Lanthanides are commonly used in upconversion applications due to their available 4f orbitals allowing for multiple electronic transitions [119]. Lanthanides have tunable absorption properties in the IR region and have shown promise when used in photovoltaic and photocatalytic applications [120]. Figure 23 shows an example of 4f-4f upconversion in a single ion (Er^3+^) using a laser source of wavelength 980 nm. The excited electrons undergo various levels of vibrational relaxation before emitting at higher energies than the original excitation wavelength [120].

In 2012, Bao et al. demonstrated upconversion photoluminescence in poly(methyl methacrylate) (PMMA) nanofibres containing lanthanide-doped nanoparticles [121]. The lanthanide-doped nanoparticles efficiently convert low-energy near-infrared (NIR) photons to high-energy visible photons. Specifically, they used ytterbium oxide and erbium oxide as their upconverting materials. The PMMA/ytterbium oxide/erbium oxide solution was electrospun using a ~20 G needle, a voltage of 20 kV, and 20 cm from tip-to-collector. The nanoparticles aggregated along the nanofibres, which was consistent with previous findings. Despite this, the upconversion emission was consistent in the nanofibres as found in powder form (emission at 523, 539, and 656 nm). The nanoparticles were capped with oleic acid and were found to be aligned along fibre axes, and the electrospun films were flexible and uniform, with minimal beads when visualized using SEM imaging. The collection drum was located 20 cm from the tip of the spinneret, and a voltage of 20 kV was used for electrospinning [121].

In another study, NaYF_4_:YB^3+^, Er^3+^ nanoparticles were incorporated into polymeric silica and electrospun. NaYF_4_:YB^3+^, Er^3+^ are reported as efficient NIR-to-visible upconverting materials, and silica has modifiable surface pores with Si-OH active bonds located on pore walls [122]. The use of multiple-lanthanide nanoparticles allows for electron transfer between atoms to produce the resulting upconverted light. The tip-to-collector distance was set at 20 cm with 10 kV of voltage supplied, and the nanofibres emitted at 522 nm, 542 nm, 655 nm, and 663 nm upon excitation with a 980 nm laser [122].

The use of upconverting nanomaterials can also be used to alter the transparency of materials owing to the ability of these materials to absorb outside of the visible spectrum [123]. In one study, upconverting nanoparticles (UCNPs) were used to develop transparent nanofibre films [124]. In this study, poly-methyl methacrylate (PMMA) was used owing to its transparent properties alongside photoluminescent nylon 6 (PA6). The upconverting materials were Y_2_O_3_, Yb_2_O_3_, and Er_2_O_3_. Upon excitation at 980 nm, the UCNP showed strong upconversion emission around 550 nm [124]. Other examples of the use of upconverting nanoparticles in electrospun nanofibres include the incorporation of Y_2_Ti_2_O_7_:Tm/Yb UCNPs in PVP [125] and Bi_2_Ti_2_O_7_:Tm^3+^/Yb^3+^ in PVP [126].

## 4. Charge Transfer Systems in Electrospun Nanofibres: Photoinduced Electron Transfer (PET)

**Figure 24:**
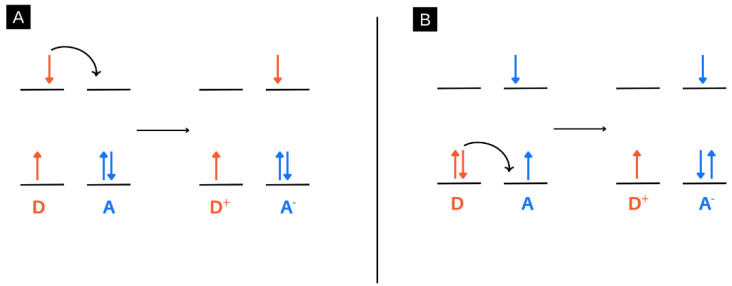
Examples of the photoinduced electron transfer (PET) process. (**A**) An electron is transferred from the LUMO of the acceptor to the LUMO of the donor. (**B**) An electron is transferred from the HOMO of the donor to the HOMO of the acceptor.

Photoinduced electron transfer (PET) is a charge transfer process after photoexcitation whereby an electron is transferred from a donor to acceptor (Figure 24). This can occur with the donor and acceptor being directly bound or by being linked via a spacer group [127]. The PET process can be used for the development of fluorescent probes that react to their environment and result in an “on” or “off” fluorescent state [128].

**Figure 25:**
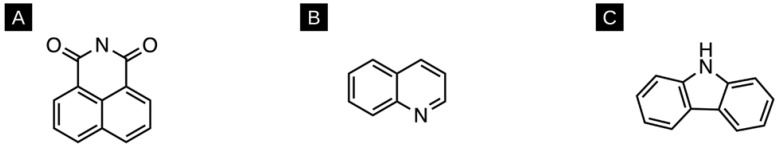
Common fluorophores used in photoinduced electron transfer processes. (**A**) Napthalimide, (**B**) Quinoline, (**C**) Carbazole.

Various fluorophores that exhibit the PET process have been explored, and some have been electrospun in nanofibres as either donors or acceptors. Examples of molecules that exhibit PET include naphthalamides, quinolines, and carbazoles, as shown in Figure 25.

### 4.1. Acceptors: Naphthalimide and Quinoline

Naphthalimides are effective for fluorescent detection, as their optical properties can be readily altered, and they are sensitive to the polarity of their surrounding environment. Naphthalimides have been used for applications as therapeutic agents and as chemical probes [129,130]. The typical core structure of the naphthalimides is shown in Table 1, but a variety of substituents can be added to the aromatic naphthalene or the N-imide resulting in a change in fluorescent properties. Basic naphthalamides and non-complex derivatives of 1,8-napthalimide typically absorb around 360 nm and emit around 440 nm [131]. However, this can shift depending on the derivative and the solvent, as shown in Table 1.

Naphthalimide fluorescence intensity is highly responsive to changes in pH [135]. This occurs due to a photoinduced electron transfer (PET) effect within the napthalamine structure. PET occurs from a receptor to the naphthalamide, and this process is extremely sensitive to analytes or pH changes, allowing for fluorescence enhancement or quenching.

Liu et al. incorporated naphthalamides into electrospun polymers to detect the pH level in water. In this study, the backbone of the polymer was poly(methylacrylate) (PMA) with a 1,8-naphthalamide and a zwitterionic side chain to ensure fluorescence and enhance hydrophilicity, respectively. The polymer was blended with PVA for electrospinning and was exposed to pH values ranging from 4–10. Under acidic conditions, the piperazine group in this derivative is protonated, and photoinduced electron transfer between the donor and naphthalamide acceptor is inhibited, which results in strong fluorescence. Conversely, under alkaline conditions, photoinduced charge transfer between the piperazine group and naphthalamide fluorophore can occur, which quenches fluorescence.

Quinoline and its derivatives are excellent fluorescent sensors owing to their high selectivity and low detection limit (at the nM or pM scale). Outside of PET, quinoline derivatives also demonstrate intermolecular charger transfer (ICT) and FRET. Typically, for PET “fluorescence on” applications, quinoline is connected to a group that contains high-energy non-bonding electrons (e.g., nitrogen). This group will transfer an electron to quinoline quenching fluorescence [136]. However, if that group is coordinated by a cation, the electron transfer process is prevented, resulting in fluorescence of quinoline [136].

**Figure 26:**
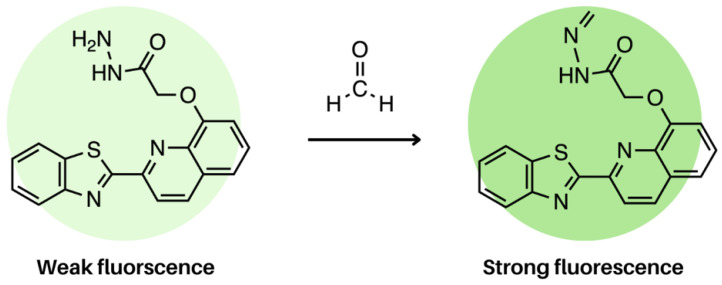
PET-induced fluorescence of 8-HQ. In the absence of formaldehyde, the fluorophore is weakly fluorescent. Upon interaction with formaldehyde, the fluorophore exhibits a five-fold increase in fluorescence intensity thanks to the PET process between the two. This fluorophore was embedded in electrospun nanofibres films as a sensor [137].

In a study done by Liu et al., 8-hydroxyquinoline (8-HQ) was electrospun into nanofibres for the detection of formaldehyde. Formaldehyde is a carcinogen and environmental pollutant that is heavily used in manufacturing. With that in mind, the detection of formaldehyde in food production is critical to avoid consumer contact. Prior to this study, detection methods relied on fluorescent probes dissolved in solvents; however, solid-state probes are non-invasive and provide a real-time signal. Therefore, this study incorporated 8-HQ in PVA and electrospun the solution at a voltage of 14 kV, 0.7 mL/h and a receiving distance of 14 cm [137]. In this case, PET naturally occurs in 8-HQ quenching fluorescence, but with the introduction of formaldehyde, electron donation from nitrogen to the quinone no longer occurs, allowing for fluorescence, as shown in Figure 26. The emission maximum of 8-HQ occurs at 467 nm, and a 5.5-fold fluorescence enhancement was observed upon the introduction of formaldehyde. Liu et al. also explored the PET process using DFT calculations, which showed that in the absence of formaldehyde, the energy level of the fluorophore (−5.997 eV) is lower than that of the aniline (−5.957 eV), which drives the PET process. In the presence of formaldehyde, the energy of the imine (−6.024 eV) is lower than that of the fluorophore, preventing PET and allowing for fluorescence [137].

### 4.2. Donors: Carbazole

Carbazole has been widely studied within oligomers, dendrimers, and polymers for applications as photoluminescent and optoelectronic materials [138]. Carbazole is inexpensive, its nitrogen moiety permits additional functionalization, and it can be readily polymerized [139]. Carbazole and its non-complex derivatives typically have three absorption peaks between 260–350 nm and emit between 300–400 nm, depending on the solvent used.

**Figure 27:**
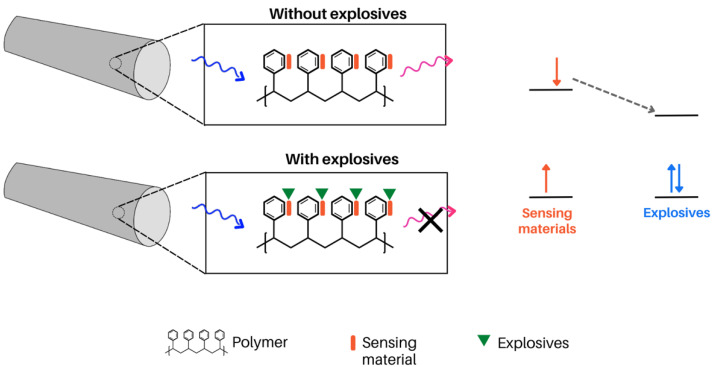
Polystyrene films with carbazole-based sensing materials (9-(Pyren-1-yl)-9H-carbazole) were used for the detection of explosives (DNT and TNT). In this example, PET occurs between the sensing material and the explosive, quenching the fluorophore when an explosive is nearby [140].

A study done by Wu et al. explored the use of photoinduced electron transfer via a carbazole derivative for the detection of explosives. Sensing applications, such as this one, rely on high-surface materials to allow for the analyte to be easily incorporated into the material and be in contact with the fluorophore. As such, Wu et al. explored the incorporation of carbazole derivatives in electrospun nanofibres for the detection of 2,4-dinitrotoluene (DNT) and trinitrotoluene (TNT). Upon interaction with explosives, a photoinduced electron transfer event occurs where the carbazole derivative donates an electron to the nitro explosive, quenching fluorescence of the carbazole (Figure 27) [140]. In this study, 9-(Pyren-1-yl)-9H-carbazole was synthesized and incorporated into both PEO and PS solutions before electrospinning. The PEO solution included 200 mg of PEO and 1 mg of PyCz in 2mL of chloroform. The solution was electrospun at a voltage of 19 kV, 1.0 mL/h flow rate, 0.99 mm needle, and a receiving distance of 15 cm. The PS solution was prepared with 200 mg PS and 5 mg PyCz in DMF. The solution was electrospun at a voltage of 15 kV, 1.0 mL/h flow rate, 0.6 mm needle, and a receiving distance of 15 cm. Both films were responsive to the presence of explosives and demonstrated a change in fluorescence [140].

## 5. Conclusions

Electrospinning is a process whereby polymeric nanofibre films are developed from solutions. These nanofibres can come in a variety of structures such as monoaxial, coaxial (core@shell), and Janus (side-by-side). More interestingly, light-harvesting materials such as metal nanoparticles, fluorophores, and quantum dots can be incorporated in these nanofibres, effectively enhancing their function. These light-harvesting materials permit various photo-driven processes to occur in the nanofibre films, including Förster resonance energy transfer (FRET), metal-enhanced fluorescence (MEF), and photoinduced electron transfer (PET). By embedding light harvesting materials in the nanofibres, functionalized films are created for a wide variety of applications including sensors, OLEDs, photovoltaic devices, drug delivery systems, etc. The opportunity for future work in this field is vast. Increased focus on exploiting the controlled confinement of light-harvesting materials in these 1D structures (for example at interfaces in core@shell fibres) and their tailored pseudo-mobility could revolutionize the function of these nanofibres towards light-driven reactivity and catalysis. Other light-driven systems can also be explored such as light-driven polymer degradation and controlled agent release for bio-compatible drug delivery.

## Figures and Tables

**Table 1:** Absorption and emission data of several napthalimide-based molecules in varying solvents.

Structure	Name	Absorbance Maxima	Emission Maxima	Solvent	Reference
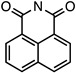	1,8-napthalimide	360 nm	440 nm	DMF	[131]
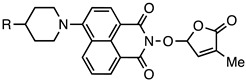	2-(4-Methyl-5-oxo-2,5-dihydrofuran-2-yloxy)-6-(4-methylpiperidin-1-yl)- benzo[de]isoquinoline-1,3-dione	402	508	dioxane	[132]
		416	521	acetonitrile	[132]
		420	528	methanol	[132]
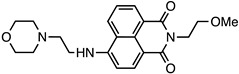	2-(2-methoxyethyl)-6-((2-morpholinoethyl)amino)-1H-benzo[de]isoquinoline1,3(2H)-dione	430	520	acetonitrile	[133]
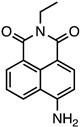	4-amino-1,8-napthalimide	450 nm	550 nm	water	[134]

## Data Availability

Not applicable.
